# Improvement of P300-Based Brain–Computer Interfaces for Home Appliances Control by Data Balancing Techniques

**DOI:** 10.3390/s20195576

**Published:** 2020-09-29

**Authors:** Taejun Lee, Minju Kim, Sung-Phil Kim

**Affiliations:** Department of Human Factors Engineering, Ulsan National Institute of Science and Technology, Ulsan 44919, Korea; xowns325@unist.ac.kr (T.L.); mjkim28@unist.ac.kr (M.K.)

**Keywords:** brain–computer interfaces (BCI), electroencephalography (EEG), P300, sampling techniques, borderline-SMOTE

## Abstract

The oddball paradigm used in P300-based brain–computer interfaces (BCIs) intrinsically poses the issue of data imbalance between target stimuli and nontarget stimuli. Data imbalance can cause overfitting problems and, consequently, poor classification performance. The purpose of this study is to improve BCI performance by solving this data imbalance problem with sampling techniques. The sampling techniques were applied to BCI data in 15 subjects controlling a door lock, 15 subjects an electric light, and 14 subjects a Bluetooth speaker. We explored two categories of sampling techniques: oversampling and undersampling. Oversampling techniques, including random oversampling, synthetic minority oversampling technique (SMOTE), borderline-SMOTE, support vector machine (SVM) SMOTE, and adaptive synthetic sampling, were used to increase the number of samples for the class of target stimuli. Undersampling techniques, including random undersampling, neighborhood cleaning rule, Tomek’s links, and weighted undersampling bagging, were used to reduce the class size of nontarget stimuli. The over- or undersampled data were classified by an SVM classifier. Overall, some oversampling techniques improved BCI performance while undersampling techniques often degraded performance. Particularly, using borderline-SMOTE yielded the highest accuracy (87.27%) and information transfer rate (8.82 bpm) across all three appliances. Moreover, borderline-SMOTE led to performance improvement, especially for poor performers. A further analysis showed that borderline-SMOTE improved SVM by generating more support vectors within the target class and enlarging margins. However, there was no difference in the accuracy between borderline-SMOTE and the method of applying the weighted regularization parameter of the SVM. Our results suggest that although oversampling improves performance of P300-based BCIs, it is not just the effect of the oversampling techniques, but rather the effect of solving the data imbalance problem.

## 1. Introduction

Noninvasive brain–computer interfaces (BCIs) utilizing P300, an event-related potential (ERP) component [1], have been extensively studied for various applications [2,3,4]. P300 component refers to a positive ERP peak that appears between 250 and 500 ms after event onset [5]. P300 is mainly elicited in an oddball paradigm consisting of frequently presented nontarget stimuli along with infrequently presented target stimuli. Target stimulus classification through P300 allows a BCI to infer and transmit the user’s intents to external devices. Farwell and Donchin created a P300-based BCIs system that enables typing letters only by brain activities [6]. Since then, P300-based BCIs have also been widely applied to control other devices such as a wheelchair, robot, and games [7,8,9,10].

P300-based BCIs can be integrated in a smart home system to control living devices with brain activity [11]. Carabalona et al. developed P300-based BCIs for controlling bulbs, doors, and speakers through a 6 × 6 matrix user interface and showed the accuracy of approximately 50% through the linear discriminant analysis (LDA) classifier [12]. Edlinger et al. built a hybrid BCI system for smart home control using both P300 and steady-state visually evoked potential (SSVEP) in a virtual reality (VR) environment. In this system, a P300-based interface was used to select a target from many alternatives and an SSVEP-based interface was used to start or stop the P300-based interface [13]. The BCI system that controlled home appliances and navigation in the VR environment achieved a range of accuracy from 83% to 100% [14].

However, the oddball paradigm used in P300-based BCIs intrinsically poses the issue of data imbalance between target stimuli and nontarget stimuli because it generates a relatively small number of ERP samples for target compared to a larger number of ERP samples for nontarget stimuli. In general, the number of data samples for nontarget is at least three times greater than that for the target. In addition, the P300 amplitude increases as the ratio of nontarget sample size to target sample size increases [15]. Such data imbalance can bias classifiers toward the class with a larger size and thus degrade classification performance by overfitting [16].

Several studies have attempted to address data imbalance problems with different approaches. First, the data level approach draws on sampling techniques by either undersampling data in larger-size classes or oversampling data in smaller-size classes. For instance, Wu et al. achieved the performance of 99.48% using one of the oversampling techniques, synthetic minority oversampling technique (SMOTE) [16], for classifying epileptic electroencephalogram (EEG) [17]. Xu et al. also showed that using SMOTE could improve the LDA classifier for P300 detection, and that oversampling was more effective than undersampling [18]. However, this study applied sampling techniques to BCIs in only 2 subjects. Second, the algorithm level approach uses an ensemble model with multiple classifiers. For instance, Shi et al. developed random undersampling bagging support vector machines (SVMs) that conducted random undersampling from nontarget data to match the number of target and nontarget data samples, trained classifiers, and detected P300 using multiple classifiers [19]. This ensemble model led to better classification performance than using single classifiers such as LDA, SVM, and convolutional neural network (CNN).

The purpose of this study is to prove the feasibility of improving the performance of P300-based BCIs using sampling techniques and to find which of the techniques provides the best performance to control various home appliances. While numerous studies have investigated P300-based BCI performance improvement through classification models using feature extraction and deep learning methods, few studies examined whether it would be plausible to improve performance by solving data imbalance. Therefore, in this study, various oversampling and undersampling techniques as well as ensemble method were explored to find the optimal sampling technique. Additionally, we investigate how sampling techniques help to build a classifier in a better way. To this end, we opt to use SVM as a classifier for BCIs rather than more complex artificial neural networks, as it is straightforward to analyze the effect of sampling on the properties of SVM such as the number of support vectors and the margin between support vectors and decision boundary.

## 2. Methods

### 2.1. Subjects

A total of 44 subjects participated in the study of controlling one of the three home appliances using BCIs (13 female, ages 18–31 years old). Fifteen of them participated in the experiment of a door lock control BCI, 15 in an electric light control BCI, and 14 in a Bluetooth speaker control BCI. They had no history of neurological illness. This study was approved by the Ulsan National Institutes of Science and Technology of Institutional Review Board (UNISTIRB-18-08-A) and all subjects gave informed consent.

### 2.2. Stimuli

The visual oddball paradigm with four or six stimuli was used to elicit P300 depending on the type of home appliance controlled. In the electric light BCI, stimuli were composed of three control (on/off/± brightness) icons as well as one dummy icon; and in the door lock BCI, stimuli consisted of two control (lock/unlock) icons and two dummy icons. In the Bluetooth speaker BCI, there were six control stimuli (play and pause/mute/volume up/volume down/next song/previous song). The ratio of the number of target to nontarget stimuli was 1:3 in the electric light and door lock BCIs, and 1:5 in the Bluetooth speaker BCI. In the case of the electric light and door lock BCIs, the stimuli were presented in the see-through user interface (UI); and in the case of the Bluetooth speaker BCI, the stimuli were presented on the LCD screen.

### 2.3. Experimental Design

**Figure 1:**
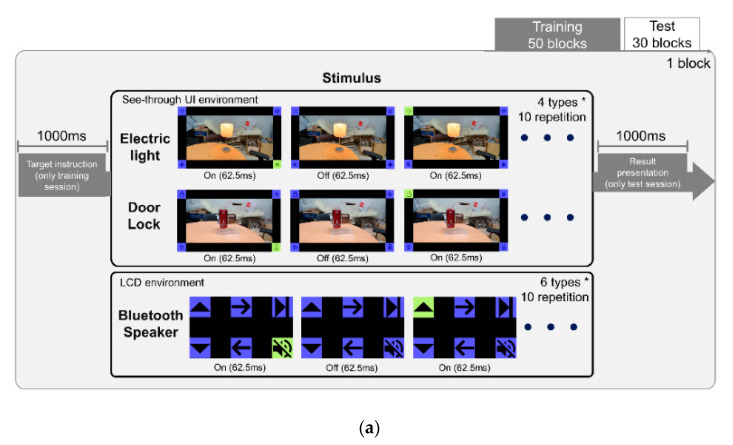

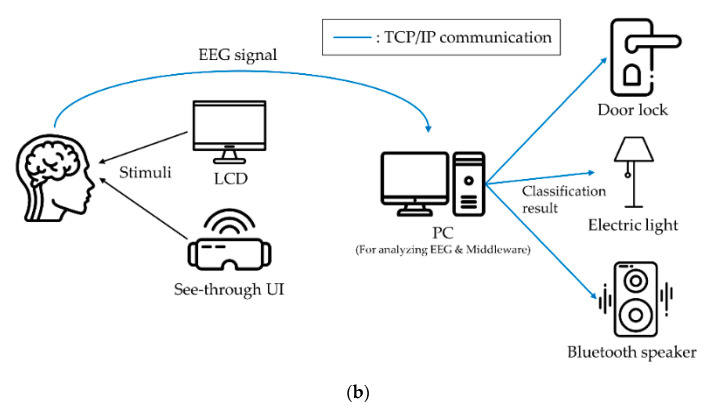
(**a**) The experimental design to build brain–computer interfaces (BCIs) for controlling three different home appliances: door lock, electric light, and Bluetooth speaker. Participants performed 50 training blocks followed by 30 test blocks. User interfaces provided to participants for controlling each home appliance are shown. (**b**) An overall diagram of the communication network between electroencephalography (EEG)-based BCIs and home appliances through TCP/IP communication.

Each experiment was composed of a training session and a test session. The training session consisted of a total of 50 blocks from which we obtained EEG data and target information to train a BCI classifier. At the beginning of each block, the target information was displayed for 1000 ms with an instruction for subjects to count the number of times the target was highlighted. Then, the main screen with all the stimuli appeared and a trial began by randomly highlighting one of the stimuli by changing its color from blue to green for 62.5 ms. Following an intertrial interval of 62.5 ms, the next trial began by randomly selecting a stimulus and highlighting it. In one block, each stimulus was highlighted exactly 10 times, so there were 40 trials per block in the electric light and door lock stimuli BCIs, and 60 trials in the Bluetooth speaker BCI. When the training session ended, we collected the subject’s EEG data and used them to train an SVM classifier (Figure 1). 

In the test session, a total of 30 blocks were performed, in which the device was controlled by BCIs in real-time. The block in the test session was designed in the same way as that in the training session. At the end of each block, the trained classifier predicted the target, and the device was controlled according to the prediction result. The result was displayed for 1000 ms. Our smart home BCI system connected middleware in PC and each home appliance via TCP/IP communication. A more detailed experimental design is described in the previous study [11].

### 2.4. EEG Processing

EEG data were acquired by an amplifier (actiCHamp, Brain Products GmBH, Gilching, Germany) with 32-channel wet electrodes, with a sampling rate of 500 Hz. Ground and reference electrodes were attached at the left and right mastoid, respectively. EEG data were preprocessed through a pipeline set in the following order: high-pass filtering (>0.5 Hz), bad channel rejection, common average re-referencing, low-pass filtering (<50 Hz), and artifact subspace reconstruction (ASR). EEG data were epoched from −200 to 600 ms after stimulus onset, and baseline correction was conducted with baseline data from −200 ms to onset. After epoching, EEG data were standardized by removing the mean and scaling to unit variance. For preprocessing, EEGLAB Toolbox [20], MATLAB (The MathWorks, Inc., Natick, MA, USA), and Python (Python Software Foundation, Beaverton, OR, USA) were used together.

### 2.5. Data Balancing

Given two-class training datasets, we first determined a majority and minority class according to the class size: majority for larger and minority for smaller class. Then, we applied the following oversampling methods to synthesize training data samples in the minority class or undersampling methods to remove training data samples in the majority class.

#### 2.5.1. Oversampling

##### Random Oversampling (ROS)

Random oversampling randomly selects and copies the minority class samples to create additional minority class instances [18].

##### Synthetic Minority Oversampling Technique (SMOTE)

SMOTE is a heuristic method of synthesizing the minority class instances using the nearest neighbors of original minority class instances [21].

The procedure is as follows:

1. Find the *K*-nearest neighbors of each minority class instance and then calculate the distances between the neighbors and instance. The value of *K* depends on the number of minority class instances to be synthesized.
2. These distances are multiplied by a random value between 0 and 1 and added to minority class instances to synthesize the new minority class instance. The equation is as follows:

(1) $$s_{j}=p+r_{j}d_{j},j=1,2,\ldots ,K,$$

where $s_{j}$ is a newly synthesized instance vector, p is an original instance vector in the minority class, $d_{j}$ is a difference vector between p and its *j*-th neighbor, and $r_{j}$ is a random value uniformly distributed between 0 and 1.

In this study, *K* is set to 5. However, since SMOTE does not consider whether the surrounding data of each instance are majority class or minority class, synthetic data can be generated in a meaningless space that does not affect the decision boundary.

##### Borderline-SMOTE (B-SMOTE)

Borderline-SMOTE is a variant of SMOTE. Since SMOTE only considers minority class instances, without taking into account adjacent majority class instances, it can create instances that interfere with building a proper classifier. To address this limitation, Han et al. proposed borderline-SMOTE, which also performs oversampling on instances of the minority class near the borderline [22].

**Figure 2:**
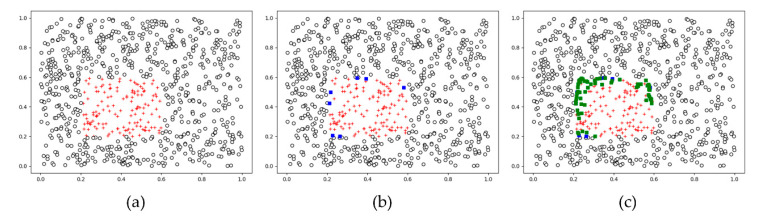
(**a**) An example of 2D binary class data with data imbalance: majority class of black circles and minority class of red crosses. (**b**) Among the minority class instances, a ‘DANGER’ set was made by selecting those instances whose neighbors are more included in the majority class than in the minority class—borderline minority instances, marked by blue squares. The number of neighbors, *K* = 5. (**c**) *K* new synthetic instances are created for each instance in the DANGER set (see the text for more details of the synthesis procedure); newly synthesized instances in the minority class are marked by green squares.

The procedure is as follows:

1. After extracting the *M*-nearest neighbors from each minority class instance, put the minority class instances into a set if more than half of the *M*-nearest neighbors of it belong to the majority class. This set is called a ‘DANGER’ set (see Figure 2b).
2. After selecting the *K*-nearest neighbors of every instance in the ‘DANGER’ set, calculate a difference vector between the instance and each of the *K*-nearest neighbors. Then, multiply the difference vector by a random value between 0 and 1 and add it to the minority data set, in the same way as the SMOTE procedures described above.

In this study, *M* and *K* are set to 10 and 5.

##### Support Vector Machine SMOTE (SVM-SMOTE)

SVM-SMOTE, another variant of SMOTE using the support vectors proposed by Nguyen et al., is similar to borderline-SMOTE, except that the support vector machine is used to define the borderline, which is equivalent to a decision boundary in SVM [23].

The procedure is as follows:

1. Find support vectors in the minority class after training a support vector machine.
2. Find the *M*-nearest neighbors of each minority class support vector.
3. Count the number of majority class instances in those *M*-nearest neighbors.
4. If the number of majority class instances is less than half, calculate a distance vector between the minority class support vector and the *K*-nearest neighbors and multiply it by a random value between 0 and 1. Then, add these *K* newly synthesized vectors to the minority class support vector, in the same way as the SMOTE procedures described above.

In this study, *M* and *K* are set to 10 and 5.

##### Adaptive Synthetic Sampling (ADASYN)

ADASYN, an adaptive oversampling method proposed to overcome the limitation of SMOTE, adjusts the number of synthetic data to be generated according to the distribution of surrounding majority class instances [24]. The procedure is as follows:

1. Determine the total number of synthetic data (*G*) to generate in the minority class using the following formula:
  (2) $$G=\beta (\left|S_{maj}\right|-\left|S_{\min}\right|),$$
  where $\left|S_{maj}\right|$ is the size of the majority class, $\left|S_{\min}\right|$ is the size of the minority class, and β∈[0,1] is a parameter used to specify the desired balance level after generation of the synthetic data.
2. Find the *K*-nearest neighbors for each instance belonging to the minority class, and calculate the portion of the majority neighbors for each instance, denoted by *γ* here, as follows:
  (3) $$\gamma (i)=\frac{1}{Z}\frac{\delta (i)}{K},i=1,\ldots ,\left|S_{\min}\right|,$$
  where δ(i) is the number of instances belonging to the majority class instances among *K*-nearest neighbors, and Z is a normalization constant that makes the sum of γ(i) equal to 1.
3. Determine the number of synthetic data (g) that need to be generated for each instance of the minority class as (if g has a decimal point, it is rounded up):
  (4) g(i)=γ(i)×G,i=1,…,K.
4. For each minority instance, generate g(i) synthetic instances using SMOTE.

In this study, β is set to 1 to equal the number of majority class and minority class data, and *K* is set to 5.

#### 2.5.2. Undersampling

Random undersampling randomly selects and removes majority class instances.

##### Neighborhood Cleaning Rule (NCR)

NCR combines the edited nearest neighbor method and the condensed nearest neighbor method [25]. The procedure is as follows:

1. First, apply the edited nearest neighbor method to the dataset. The edited nearest neighbor method removes the instances in the majority class if more than half of the *K*-nearest neighbors of an instance in the majority class do not belong to the majority class.
2. Then, the condensed nearest neighbor method is applied to the data set with noise removed through the edited nearest neighbor method. This method removes a majority class instance if its closest neighbor belongs to the majority class or not if the closest neighbor belongs to the minority class.

In this study, *K* is set to 3.

##### Tomek’s Links (Tomek)

Tomek’s link method is an undersampling technique that removes majority class instances from a Tomek’s link. The procedure is as follows:

1. Include all minority class data in the new training data set.
2. Find the Tomek’s links where the two data with different classes are closest to each other.
3. Include majority class data that does not correspond to Tomek’s link in the new training data set.

More details can be found in [26].

##### Weighted Undersampling Bagging (WUS)

One way to conduct undersampling in the context of classification is to divide the majority class data into multiple subsets, build classifiers for individual subsets, and combine classification outcomes. For instance, in Kundu and Ari’s study [27], the majority class data were divided into multiple subsets, and SVM model was built for each subset. Then, they combined the classification output of each classifier in each subset by assigning weights to those outputs according to the cross-validation results; a more non-negative weight for higher cross-validation accuracy with the sum of the weights equal to 1.

In this study, we benchmarked Kundu and Ari’s approach with the procedure given below:

1. Divide randomly shuffled nontarget data into several subsets: 3 subsets for door lock and electric light, and 5 subsets for Bluetooth speaker.
2. Pair each nontarget subset with the target dataset and train an SVM classifier for each pair.
3. Calculate the training accuracy.
4. Determine a weight to be assigned to each pair. For 4-class cases (door lock and electric light), the weights are determined as 1/6, 2/6, or 3/6 for the pairs with the lowest to the highest training accuracy. For 6-class cases (Bluetooth), the weights are determined as 1/15, 2/15, 3/15, 4/15, or 5/15 for the pairs with the lowest to the highest training accuracy.
5. Classify a new EEG data using each SVM that produces the target probability for each stimulus. Let $p_{ij}$ be the target probability for the *j*-th stimulus from the SVM trained with the *i*-th subset, where *j* = 1, …, *C* (*C* is the number of stimuli, i.e., 4 or 6) and *i* = 1, …, *K* (*K* is the number of subsets, i.e., 3 or 5).
6. Multiply the target probability values by the weights assigned to each SVM. Let *w_i_* be the weight assigned to the *i*-th subset. Then, the multiplication yields $w_{i}p_{ij}$ for *i* = 1, …, *K*.
7. Sum these weighted target probability values over all SVMs for each stimulus and choose the stimulus with the highest value as a target:
  (5) $$\mathrm{Estimated}\;\mathrm{target}=\underset{j}{\arg\max}\sum_{i=1}^{K}w_{i}p_{ij}.$$

### 2.6. Classification

#### 2.6.1. EEG Feature Extraction

The features specified as inputs of the support vector machine in this study were composed of the ERP amplitudes of every EEG channel (excluding bad channels). The ERP amplitudes were collected from 100~600 ms after stimulus onset, which generated 250 data points per channel with the 500-Hz sampling rate. Since there were 50 training blocks, the door lock and electric light experiments produced 50 target class samples and 150 nontarget class samples, whereas the Bluetooth speaker experiment produced 50 target class samples and 250 nontarget class samples.

#### 2.6.2. Classification Algorithm

In this study, we used SVM for classification, which has been widely used for BCIs [28,29,30]. SVM has been successfully applied to high-dimensional classification problems and helps to avoid unnecessary additional computational steps such as feature selection or dimensionality reduction [31]. To determine the classification algorithm, we applied different classification algorithms other than SVM to our P300 data. In particular, we examined the use of linear discriminant analysis (LDA) and decision tree (DT). A comparison analysis showed that classification accuracy was 46.59% with decision tree, 83.56% with LDA, and 86.06% with SVM with the original P300 data. Therefore, we chose to use SVM with the highest accuracy and investigated if we could further improve its performance with oversampling. Another reason to opt for SVM over other methods was because we expected that sampling would be more effective for SVM since it might help with determining support vectors by adding more samples to the decision boundary.

SVM aims to maximize the margin defined by distance between the decision boundary and the support vectors of each class. Considering overlapping between classes, we used soft margin SVM, whose objective function is given by

(6) $$\underset{w,b,\xi }{\min}\frac{1}{2}w^{T}w+C\sum_{i=1}^{l}\xi_{i},$$

subject to $y_{i}(w^{T}\phi (x_{i})+b)\geq 1-\xi_{i},\xi_{i}\geq 0,i=1,\ldots ,l,$

$$y_{i}=\{\begin{array}{c} -1\;\left(if\;the\;class\;of\;y\;is\;negative\right)\\ 1\;\left(if\;the\;class\;of\;y\;is\;positive\right) \end{array}.$$

In this study, the regularization parameter C is fixed to 1 without optimization. 

The process of training and testing SVM is as follows:

1. Train the binary SVM that classifies target and nontarget stimuli using the training session data.
2. For each test block data, calculate the target probability of each stimulus by the trained SVM model.
3. The stimulus with the highest target probability is classified as the target stimulus, and the remaining stimuli are classified as nontarget stimuli.

#### 2.6.3. Classification Performance Evaluation

To evaluate classification performance for each smart home BCI, accuracy was defined as the percentage of the test blocks in which the predicted target matches the actual target. We also calculated recall, precision, and *F*_1_-score based on the confusion matrix given by Table 1.

Recall, precision, and F1-score are calculated from Table 1 as follows:

(7) $$\mathrm{Recall}=\frac{TP}{TP+FN},$$

(8) $$\mathrm{Precision}=\frac{TP}{TP+FP},$$

(9) $$F1\;\mathrm{score}=2\times \frac{Precision\times Recall}{Precision+Recall}.$$

The higher value of recall in BCIs means that the classification algorithm can successfully detect a target stimulus whenever it is presented. The higher value of precision means that when the classification algorithm classifies a stimulus as a target, it is more likely a real target stimulus. As an example, if the recall is high but precision is low, the classification algorithm tends to classify nontarget stimuli as well as target ones as targets, showing biased classification toward the target. *F*_1_-score overcomes this possible trade-off between recall and precision by using the harmonic mean of these two metrics. *F*_1_-score can more accurately describe the performance of classification algorithms especially when class sizes are unbalanced. 

The information transfer rate (ITR), which has been widely used for evaluating BCI performance, indicate the amount of information delivered from the brain to external devices by BCIs for a certain period. Since BCIs need to be able to send a large amount of information to devices as quickly as possible, BCI researches have been focused on increases in ITR, which requires the increase of accuracy, the number of target classes, and/or the reduction of processing time. ITR is calculated as follows:

(10) $$\mathrm{ITR}(bits/\min)=\frac{\log_{2}N+P\log_{2}P+(1-P)\log_{2}[(1-P)/(N-1)]}{T},$$

where *N* is the number of target classes, *P* is classification accuracy, and *T* is the time elapsed to control devices (in the unit of minute). For instance, for the BCIs built in this study, it took 1 s to select one of the given control menus by a BCI in each test block.

## 3. Results

### 3.1. Effects of Balancing on BCI Accuracy

**Figure 3:**
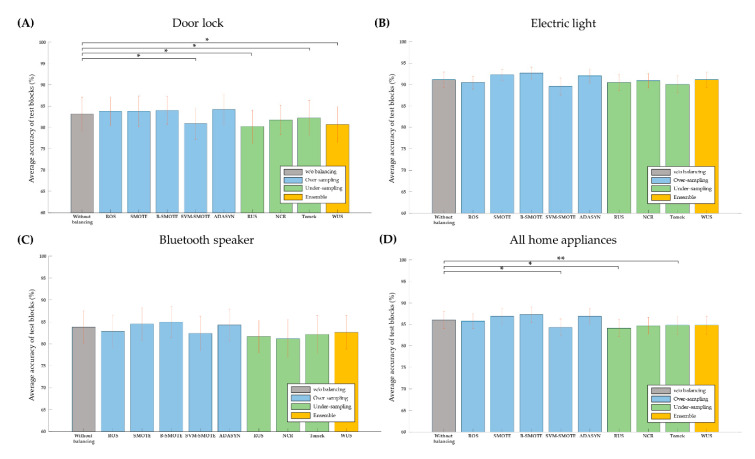
Average accuracy of controlling each home appliance using P300-based BCIs without balancing as well as various over-, and undersampling techniques for balancing. See the text for details. Accuracy for controlling for each home appliance as well as total accuracy is shown: (**A**) door lock, (**B**) electric light, (**C**) Bluetooth speaker, and (**D**) sum of them. Red vertical bars denote standard error of mean and * marks denote statistical significance (*p* < 0.05), and ** mark denote statistical significance (*p* < 0.005).

Overall, oversampling improved the accuracy of P300-based BCIs while undersampling worsened it. For individual home appliances, using the oversampling technique of ADASYN resulted in the highest accuracy (84.22% ± 13.89), while all the undersampling techniques lowered accuracy for the door lock BCI (Figure 3A). Notably, SVM-SMOTE also reduced accuracy. Using borderline-SMOTE produced the highest accuracy for the electric light BCI (92.67% ± 5.52) and for the Bluetooth speaker BCI (85% ± 13.25) (Figure 3B,C). Once again, the undersampling techniques did not improve accuracy in these BCIs. Combining all three home appliances’ BCIs together, borderline-SMOTE showed the highest accuracy (87.27% ± 11.46), while SVM-SMOTE, RUS, and Tomek techniques reduced accuracy (Figure 3D).

Notwithstanding accuracy results, all the sampling techniques increased *F*_1_-scores (Table 2) compared to the normal classification without balancing. Specifically, most over- and undersampling techniques increased recall at the expense of decreases in precision. These results imply the effect of sampling techniques to partially correct the bias of classification toward the nontarget class due to data imbalance.

### 3.2. Performance Improvement of Poor Performers

**Figure 4:**
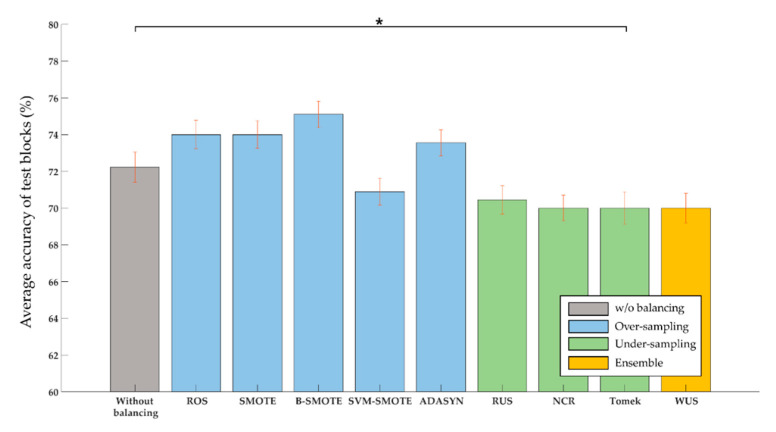
Total accuracy over all three home appliances in poor BCI performers without balancing as well as with various sampling techniques. Red vertical bars denote standard error of mean and * marks denote statistical significance (*p* < 0.05).

We investigated whether the sampling techniques could improve the performance of poor performers who showed lower accuracy than the average (86.06%) across all the home appliances in the normal classification without sampling. A total of 15 out of 44 subjects were determined as poor performers. We found that all the oversampling techniques except SVM-SMOTE improved the accuracy of poor performers (Figure 4). In particular, using borderline-SMOTE showed the largest improvement (2.89%) in accuracy. On the contrary, the undersampling techniques reduced accuracy by approximately 2%.

### 3.3. Information Transfer Rate (ITR) Improvement

**Figure 5:**
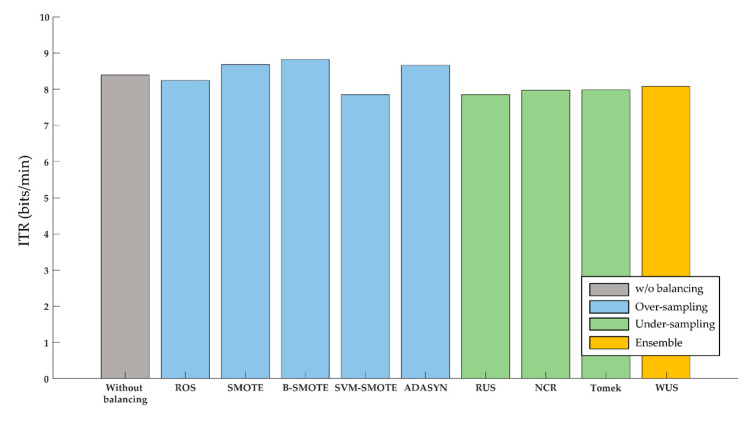
Information transfer rate (ITR) of BCIs over all three home appliances without balancing as well as with various sampling techniques.

Improvement of accuracy also gave rise to increases in ITR. For individual home appliances, ADASYN resulted in the highest ITR (3.71 bpm) for the door lock BCI; and borderline-SMOTE yielded the highest ITR for both the electric light BCI (11.33 bpm) and the Bluetooth speaker BCI (11.48 bpm). Combining all the three home appliances, borderline-SMOTE produced the highest ITR (8.82 bpm) (Figure 5). On the contrary, the undersampling techniques did not improve ITR. 

### 3.4. Effect of Borderline-SMOTE on SVM

We further investigated how the oversampling affected SVM, especially with borderline-SMOTE, which showed the best performance. In the SVM trained without oversampling, the average number of support vectors in the target class was 40.7, and that in the nontarget class was 63.11. When the SVM was trained with oversampling by borderline-SMOTE, the number of support vectors in the target class increased to an average of 56.98. As such, borderline-SMOTE increased the ratio of the number of support vectors in the target class to that in the nontarget class from 0.67 to 0.83. Moreover, the average relative distance between the support vectors to the decision boundary, which represented the margin that SVM aimed to maximize, increased by 6.05% when borderline-SMOTE was used.

### 3.5. A Comparison with the Models with Revised Regularization Parameter

The problem of data imbalance can also be handled through the regularization parameter (C) of the SVM classifier. The value of *C* determines the amount of penalty given for errors. As the value of C increases, the margin width decreases, resulting in a stricter SVM that does not tolerate error much. We adjusted the value of C by multiplying the inversely proportional weight of the number of data samples in each class. It yielded a classification accuracy of 86.82% on average, which was not significantly different from that of borderline-SMOTE.

### 3.6. Effect of the Ratio between Target and Nontarget Data on Performance

**Figure 6:**
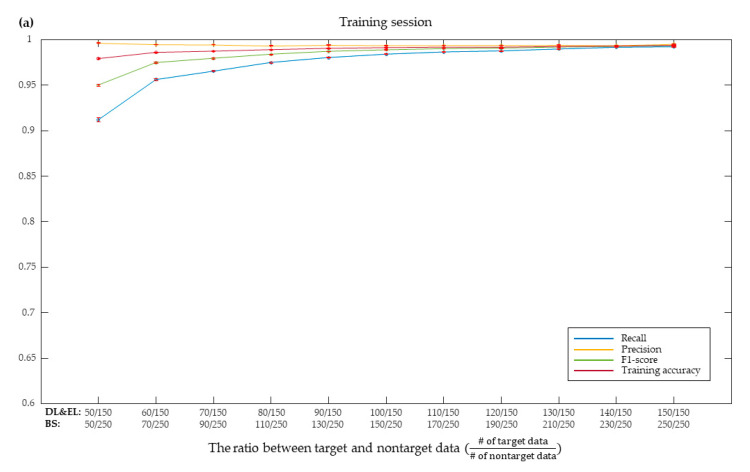

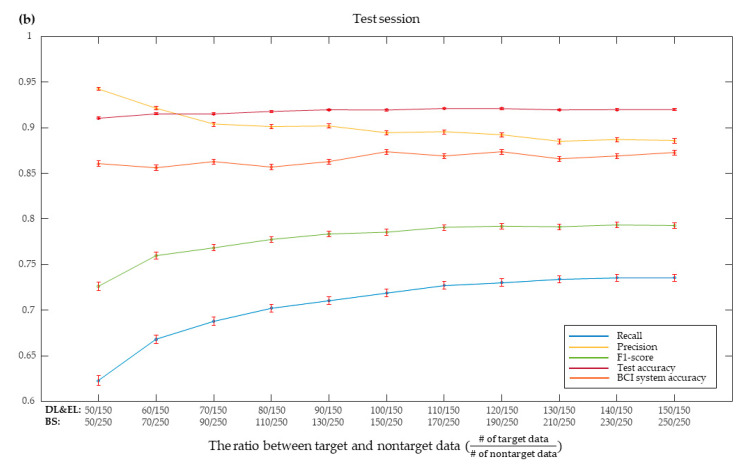
Performance of raw model with borderline-SMOTE according to target and nontarget data ratio during training (**a**) and testing (**b**), respectively. DL: Door lock, EL: Electric light, BS: Bluetooth speaker. Training accuracy is the result of binary classification that classifies target and nontarget. BCI system accuracy is the accuracy to select a target among stimuli presented through target probability.

Performance changes according to the ratio of target sample size to nontarget sample size were examined for the SVM with borderline-SMOTE. The ratio varied from 1/3 to 1 for the door lock and electric light BCIs and 1/5 to 1 for the Bluetooth speaker BCI, respectively. The three training performance measures—training accuracy, recall, and *F*_1_-score—monotonically increased as the ratio approached 1, where recall showed the greatest increase. On the other hand, precision slightly decreased as the ratio increased (Figure 6a). All the measures converged to certain values as the ratio became close to 1.

This trend was similar for the test performance. As the target ratio increased, the three measures of test accuracy, recall, and *F*_1_-score increased while precision decreased (Figure 6b). Yet, the extent to which F1-score increased was not as great as in the case of training, because precision decreased more as the ratio increased in the test. Note that we also added the accuracy of the BCIs—which was determined based on the target probability—to binary classification accuracy for every stimulus. To examine how the effect of the ratio on classification measures was related to BCI accuracy, we calculated linear correlation coefficients between each classification measure and BCI accuracy (Table 3). It revealed that while decreases in precision by increases in the ratio were not much correlated with BCI accuracy for training, those decreases in precision were more influential to BCI accuracy for test. 

## 4. Discussion

This study addressed the data imbalance problem inevitably generated by the oddball paradigm used in P300-based BCIs by employing sampling techniques. We applied the sampling techniques to a set of P300-based BCIs built for controlling home appliances including door lock, electric light, and Bluetooth speaker. Among over-, and undersampling techniques, classifiers in the BCIs could benefit from the oversampling approaches. Specifically, the borderline-SMOTE oversampling technique provided the most improvement in BCI accuracy (1.21% on average). This also led to increases in ITR by 0.44 bpm. Note that we also checked whether oversampling could improve the performance of the other classifiers, but little improvement was achieved in LDA or decision tree. From the in-depth analysis of the effect of borderline-SMOTE on SVM, we found that applying borderline-SMOTE increased the number of support vectors in the minority class (i.e., target class in our case), as reported in the previous study [32], as well as the relative distance between the support vectors and the decision boundary (i.e., margin). We suspect that these changes in SVM might allow for better performance in BCIs.

In line with the previous study [18], we also observed that the oversampling technique increased BCI performance while the undersampling techniques did not. We speculate that undersampling can disturb a-priori probability of the training set and thus distort the posterior distribution [33]. In addition, it is expected that the performance of the classification model decreases as the number of training samples decreases.

It was more effective when borderline-SMOTE was utilized on poor performers. It is likely that ERP data in poor performers would be more intermingled between the target and nontarget classes, making it difficult to draw a decision boundary. Then, borderline-SMOTE might help to find the decision boundary relatively more clearly by creating synthetic data (and support vectors as well) around the decision boundary. This resulted in 2.89% performance improvement in poor performers, which was more than twice the overall average accuracy gain of 1.21%.

For the BCIs built in this study, borderline-SMOTE yielded the best performance improvement among other oversampling techniques, such as SMOTE, SVM-SMOTE, and ADASYN. It was unexpected, however, why SVM-SMOTE did not help even with the use of SVM as a classifier. Since SVM-SMOTE performs oversampling using support vectors, when combined with SVM as a classifier, it may not induce a significant change in the decision boundary. However, borderline-SMOTE or ADASYN may enable to build a more general decision boundary because it oversamples minority class instances that have more than half of the majority class nearest neighbors.

In addition, there is no performance difference with SVM using a weighted regularization parameter to solve the data imbalance problem. However, the SVM model that optimizes regularization parameters showed significantly lower performance (85.38%). It means that solving the data imbalance problem by any method can improve the performance of P300-based BCIs, not only oversampling techniques.

Compared to our previous study [8], this study demonstrated that the door lock BCI improved the average accuracy by 5.3% and the average ITR by 0.22 bpm; and that the electric light BCI improved the average accuracy by 12.67% and the average ITR by 4.06 bpm [11] (note that the Bluetooth BCI was not analyzed in the previous study).

There are, however, some limitations to this study. First, the techniques used in this study showed limitations in performance improvement. Other sampling techniques not covered in this study might be effective. In the smart home P300-based BCIs, attempting to solve data imbalance problem and improve performance using oversampling did not have the expected effect. Second, in this study, only data balance was considered. In addition, there may be other feature extraction methods, feature engineering methods such as feature selection, and classification models that can improve performance. Third, the comparison focuses on performance such as accuracy and ITR. To use BCI in real life, computational costs must be considered. However, it will not be covered in detail in this study and will be covered in future studies. Fourth, the performance improvement demonstrated in this study was obtained through offline studies. To validate the approach in practice, we should demonstrate the effect of sampling techniques in real-time BCIs. Moreover, it is necessary to develop a technique to solve the data imbalance problem suitable for P300-based BCIs.

While recent developments of over-, and undersampling methods have allowed the effective training of machine learning methods, little is known about whether such sampling could also enhance the performance of P300-based BCIs, which inherently possesses a data imbalance issue due to the oddball paradigm. Moreover, no attempt has been made to compare a variety of over-, and undersampling techniques for enhancing P300-based BCIs. To address the question, this study investigated the effects of a wide array of over-, and undersampling techniques on a P300-based BCI and showed a plausibility of performance improvement in P300-based BCIs by addressing the data imbalance issue based on either parameter optimization in classification methods (if available) or oversampling of training data. As such, we anticipate that the present study may offer an insight on how to deal with data imbalance issues in BCIs.

## Figures and Tables

**Table 1:** Confusion matrix.

		Actual	
		Target	Nontarget
**Predicted**	**Target**	True Positive (TP)	False Positive (FP)
	**Nontarget**	False Negative (FN)	True Negative (TN)

**Table 2:** Recall, Precision, F1-score results for each sampling method (support vector machine, SVM). Random oversampling—ROS; synthetic minority oversampling technique—SMOTE; borderline-SMOTE—B-SMOTE; adaptive synthetic sampling—ADASYN; random undersampling—RUS; neighborhood cleaning rule—NCR; weighted undersampling bagging—WUS.

		w/o Balancing	ROS	SMOTE	B-SMOTE	SVM-SMOTE	ADASYN	RUS	NCR	Tomek	WUS
All home appliances	Recall	0.62	0.75	0.73	0.74	0.74	0.74	0.84	0.70	0.63	0.87
	Precision	0.95	0.85	0.88	0.88	0.84	0.87	0.71	0.87	0.94	0.75
	F1-score	0.75	0.80	0.80	0.80	0.79	0.80	0.77	0.78	0.76	0.81

**Table 3:** Correlation of BCI system accuracy with other performance indices. Corrcoef: Correlation Coefficient. Accuracy is the result of binary classification that classifies target and nontarget.

	Training Session				Test Session			
	Recall	Precision	F1-Score	Accuracy	Recall	Precision	F1-Score	Accuracy
Corrcoef	0.60	−0.21	0.59	0.65	0.68	−0.65	0.63	0.65
*p*-value	0.05	0.53	0.06	0.03	0.02	0.03	0.04	0.03
